# Data on gender and subgroup specific analyses of omega-3 fatty acids in the Ludwigshafen Risk and Cardiovascular Health Study

**DOI:** 10.1016/j.dib.2016.07.051

**Published:** 2016-08-03

**Authors:** Marcus E. Kleber, Graciela E. Delgado, Stefan Lorkowski, Winfried März, Clemens von Schacky

**Affiliations:** a Vth Department of Medicine (Nephrology, Hypertensiology, Endocrinology, Diabetology, Rheumatology), Medical Faculty Mannheim, Heidelberg University, Mannheim, Germany; b Competence Cluster of Nutrition and Cardiovascular Health (nutriCARD), Halle-Jena-Leipzig, Germany; c Institute of Nutrition, Friedrich Schiller University Jena, Germany; d Clinical Institute of Medical and Chemical Laboratory Diagnostics, Medical University Graz, Graz, Austria; e Synlab Academy, Synlab Holding Deutschland GmbH, Mannheim, Germany; f Omegametrix GmbH, Martinsried, Germany; g Department of Preventive Cardiology, Medizinische Klinik und Poliklinik I, Ludwig Maximilians University, Munich, Germany

## Abstract

This paper contains additional data related to the research article “Omega-3 fatty acids and mortality in patients referred for coronary angiography – The Ludwigshafen Risk and Cardiovascular Health Study” (Kleber et al., in press) [1]. The data shows characteristics of the Ludwigshafen Risk and Cardiovascular Health (LURIC) study according to tertiles of omega-3 fatty acids as well as stratified by gender. The association of proportions of omega-3 fatty acids measured in erythrocyte membranes with different causes of death is investigated with a special focus on modeling the association of EPA with mortality in a nonlinear way. Further, the association of omega-3 fatty acids with all-cause mortality adjusted for high-sensitive C-reactive protein as a marker of systemic inflammation is examined as well as the association of EPA with cause-specific death.

**Specifications Table**

**Table:** 

Subject area	Medicine
More specific subject area	Cardiovascular diseases
Type of data	Table, graph, figures
How data was acquired	Erythrocyte omega-3 fatty acid proportions were measured at baseline in 3259 participants of the Ludwigshafen Risk and Cardiovascular Health Study (LURIC) using the HS-Omega-3 Index method. Associations of omega-3 fatty acid proportions with mortality were investigated using Cox proportional hazards regression.
Data format	Analyzed
Experimental factors	Fasting blood samples were obtained by venipuncture at study entry. Fatty acid methyl esters were generated from erythrocytes that had been stored at −80 °C by acid transesterification.
Experimental features	Erythrocyte fatty acid composition was analyzed according to the HS-Omega-3 Index technology [2]. Fatty acid methyl esters were analyzed by gas chromatography using a GC2010 gas chromatograph (Shimadzu, Duisburg, Germany) equipped with a 100-m SP2560 column (Supelco, Bellefonte, PA) and using hydrogen as carrier gas. Fatty acids were identified by comparison with a standard mixture of fatty acids characteristic of erythrocytes. Results are presented as a percentage of total identified fatty acids after response factor correction.
Data source location	Ludwigshafen Heart Center in South-West Germany
Data accessibility	Data is within this article.

**Value of the data**

• Gender-stratified analysis allows the identification of gender-specific effects of omega-3 fatty acids.
• Examination of nonlinear effects on mortality risk is important to define safe reference values.
• Investigation of cause-specific mortality might provide hints to possible pathways affected by omega-3 fatty acids.

## 1 Data

**Fig. 1:**
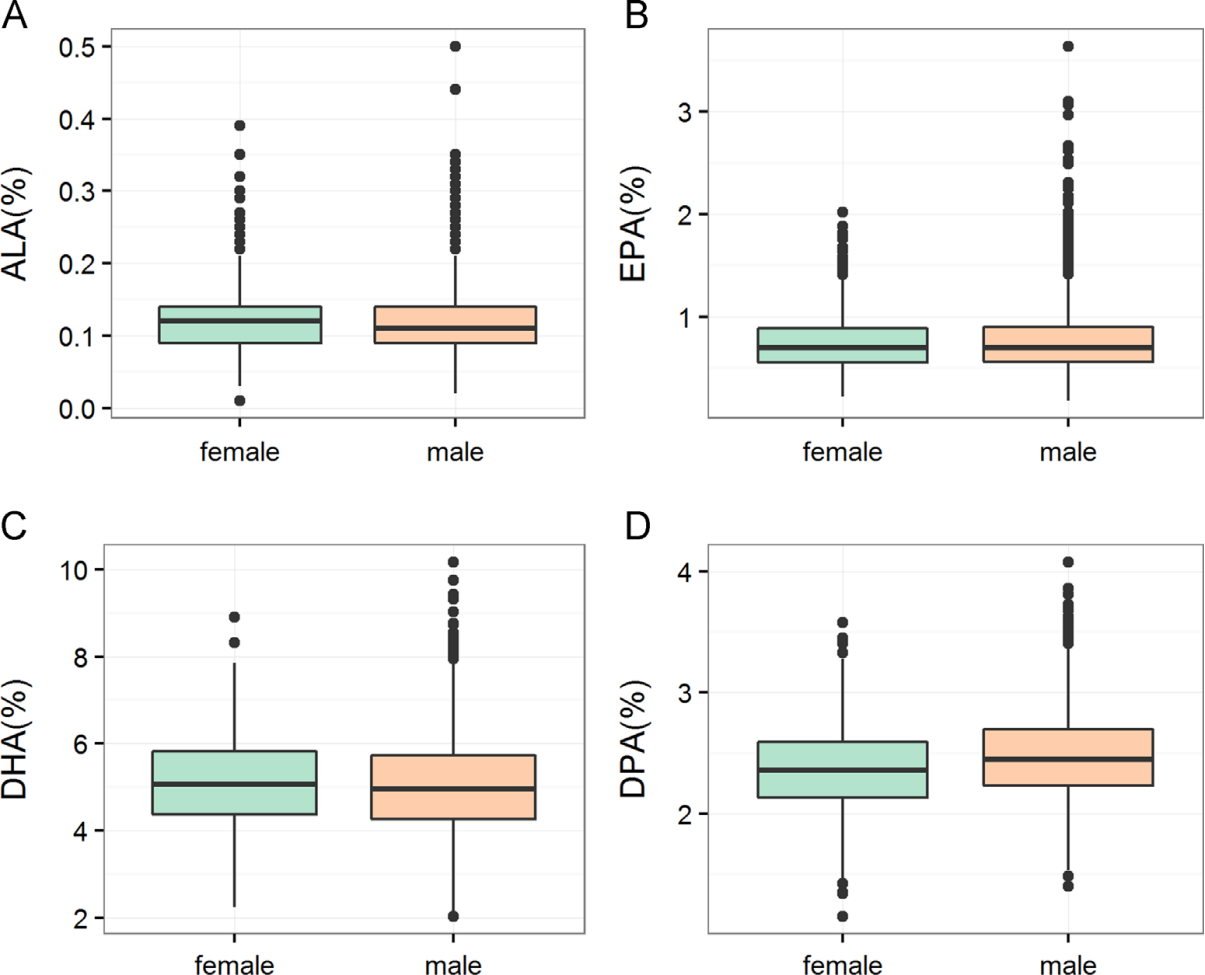
Proportions of omega-3 fatty acids in men and women. Box plots showing the distribution of ALA (A), EPA (B), DHA (C) and DPA (D) in LURIC stratified by gender. Boxes represent the interquartile ranges (IQR), median values are shown as black lines. Whiskers extend to the data point closest to a distance 1.5 times the IQR away from the median. ALA: α-linolenic acid; EPA: eicosapentaenoic acid; DHA: docosahexaenoic acid; DPA: docosapentaenoic acid.

**Fig. 2:**
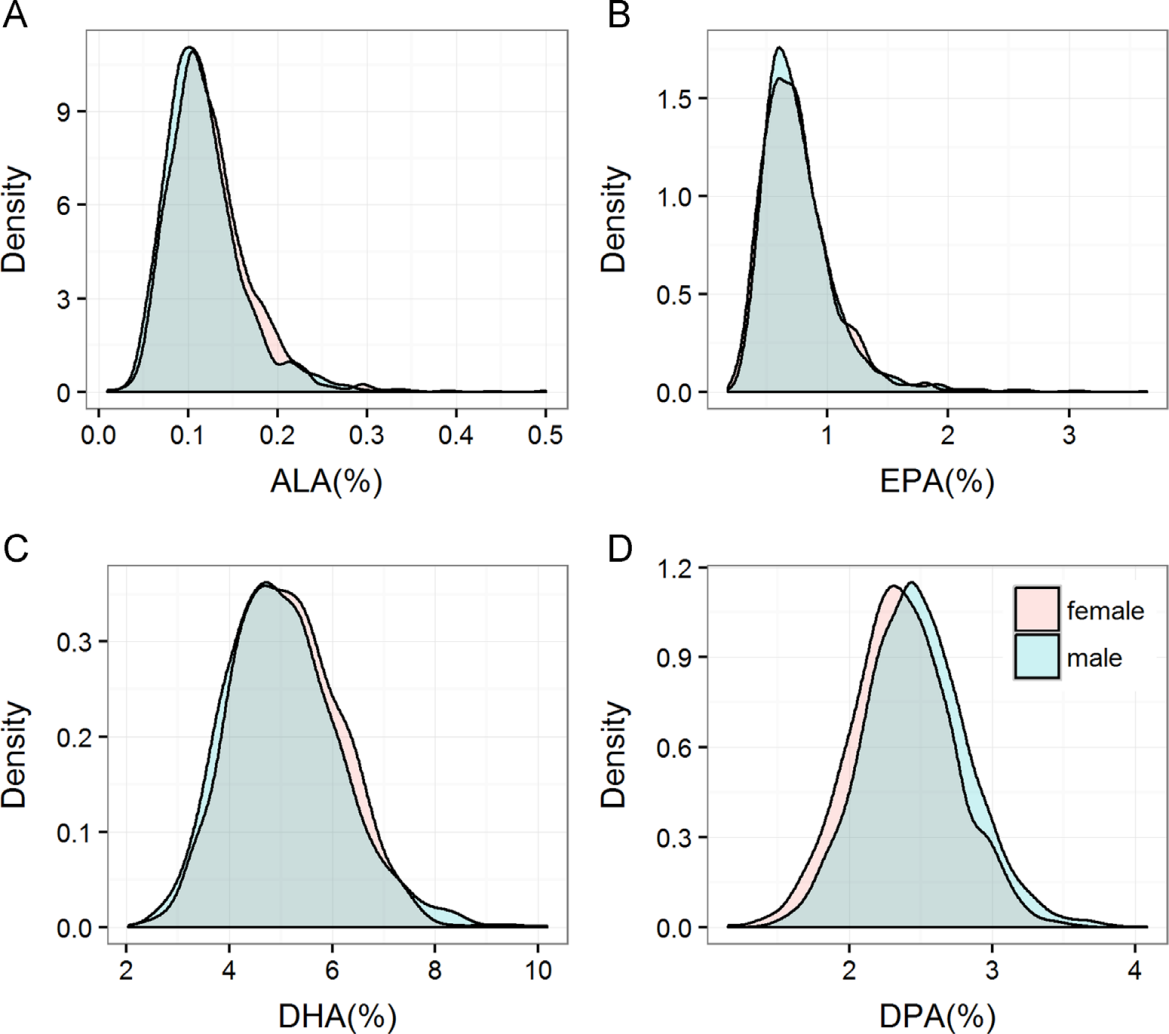
Density distribution of omega-3 fatty acid levels in men and women. Density plots showing the density distributions of ALA (A), EPA (B), DHA (C) and DPA (D) stratified by gender. ALA: α-linolenic acid; EPA: eicosapentaenoic acid; DHA: docosahexaenoic acid; DPA: docosapentaenoic acid.

**Fig. 3:**
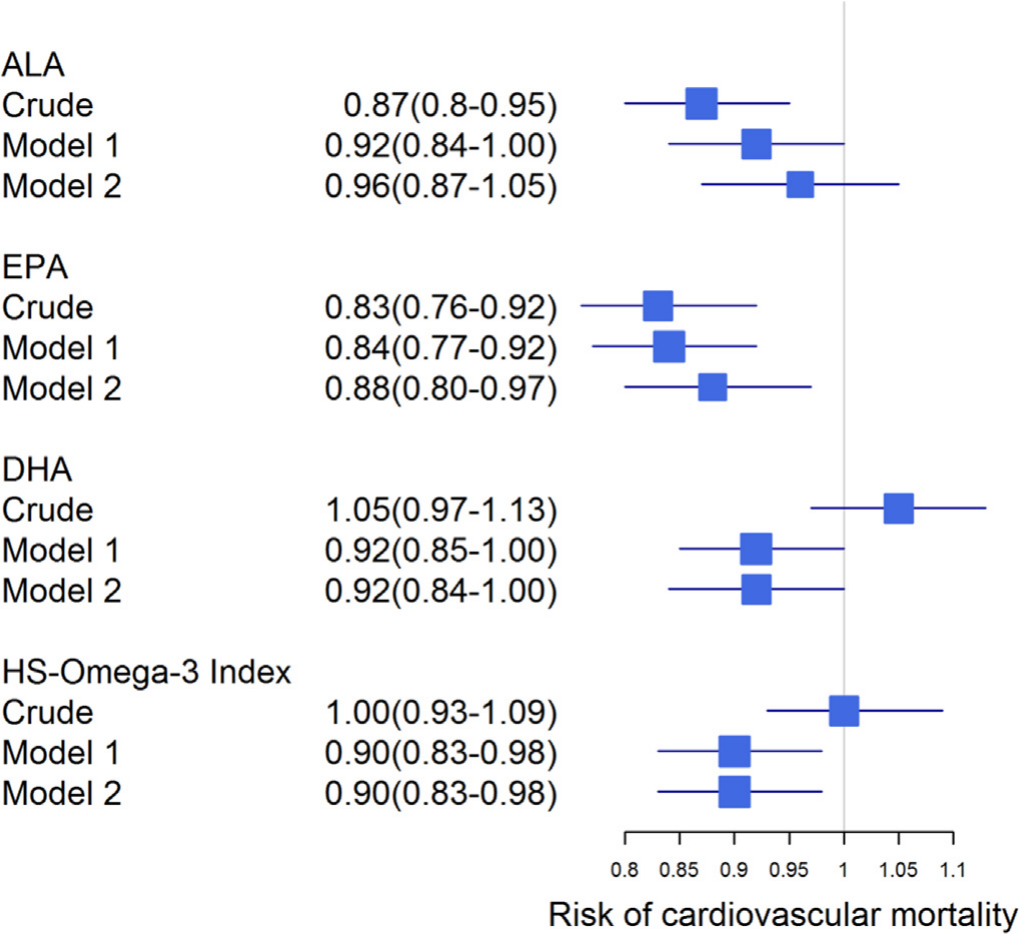
Association of omega-3 fatty acids with cardiovascular mortality. Forest plot showing the risk of CVM per 1-SD increase in omega-3 fatty acids. Hazard ratios and 95% CI were calculated for ALA, EPA, DHA and the HS-Omega-3 Index by Cox regression. Model 1: adjusted for age and gender; model 2: additionally adjusted for BMI, LDL-C, HDL-C, logTG, hypertension, diabetes, smoking, alcohol consumption, physical exercise and lipid lowering therapy. ALA: α-linolenic acid; EPA: eicosapentaenoic acid; DHA: docosahexaenoic acid; DPA: docosapentaenoic acid; TG: triglycerides.

**Fig. 4:**
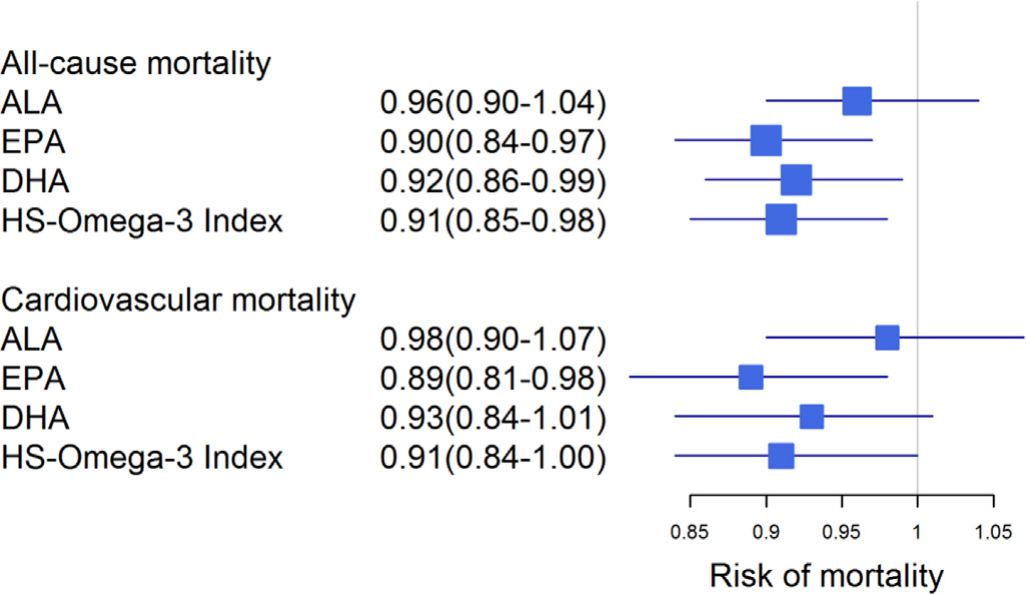
Association of omega-3 fatty acids with mortality additionally adjusted for hsCRP. Forest plot showing the risk of mortality per 1-SD increase in omega-3 fatty acids. Hazard ratios with 95% confidence intervals were calculated by Cox regression adjusted for age and gender, BMI, LDL-C, HDL-C, logTG, hypertension, diabetes, smoking, alcohol consumption, physical exercise, lipid lowering therapy and log hsCRP.

**Fig. 5:**
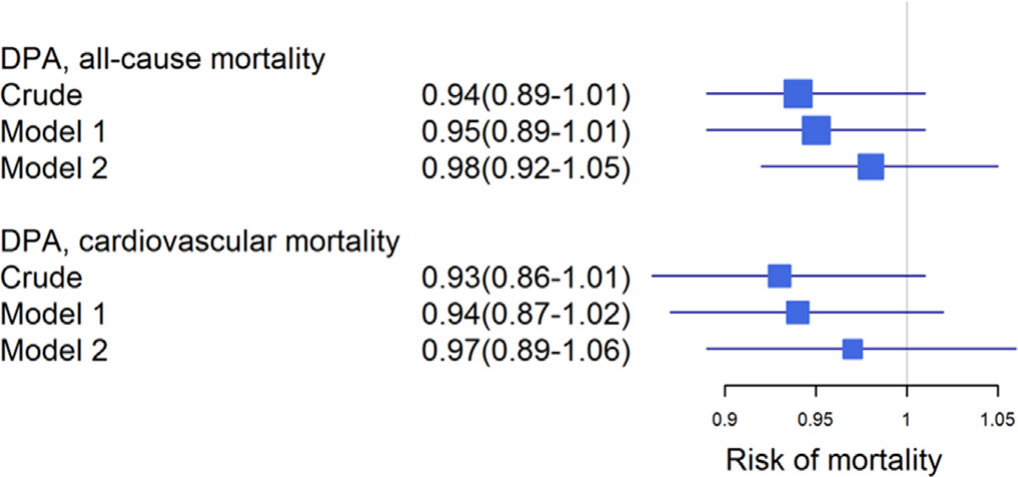
Association of DPA with mortality. Forest plot showing the risk of all-cause mortality and CVM per 1-SD increase in DPA. Model 1: adjusted for age and gender; model 2: additionally adjusted for BMI, LDL-C, HDL-C, logTG, hypertension, diabetes, smoking, alcohol consumption, physical exercise and lipid lowering therapy. DPA: docosapentaenoic acid.

**Fig. 6:**
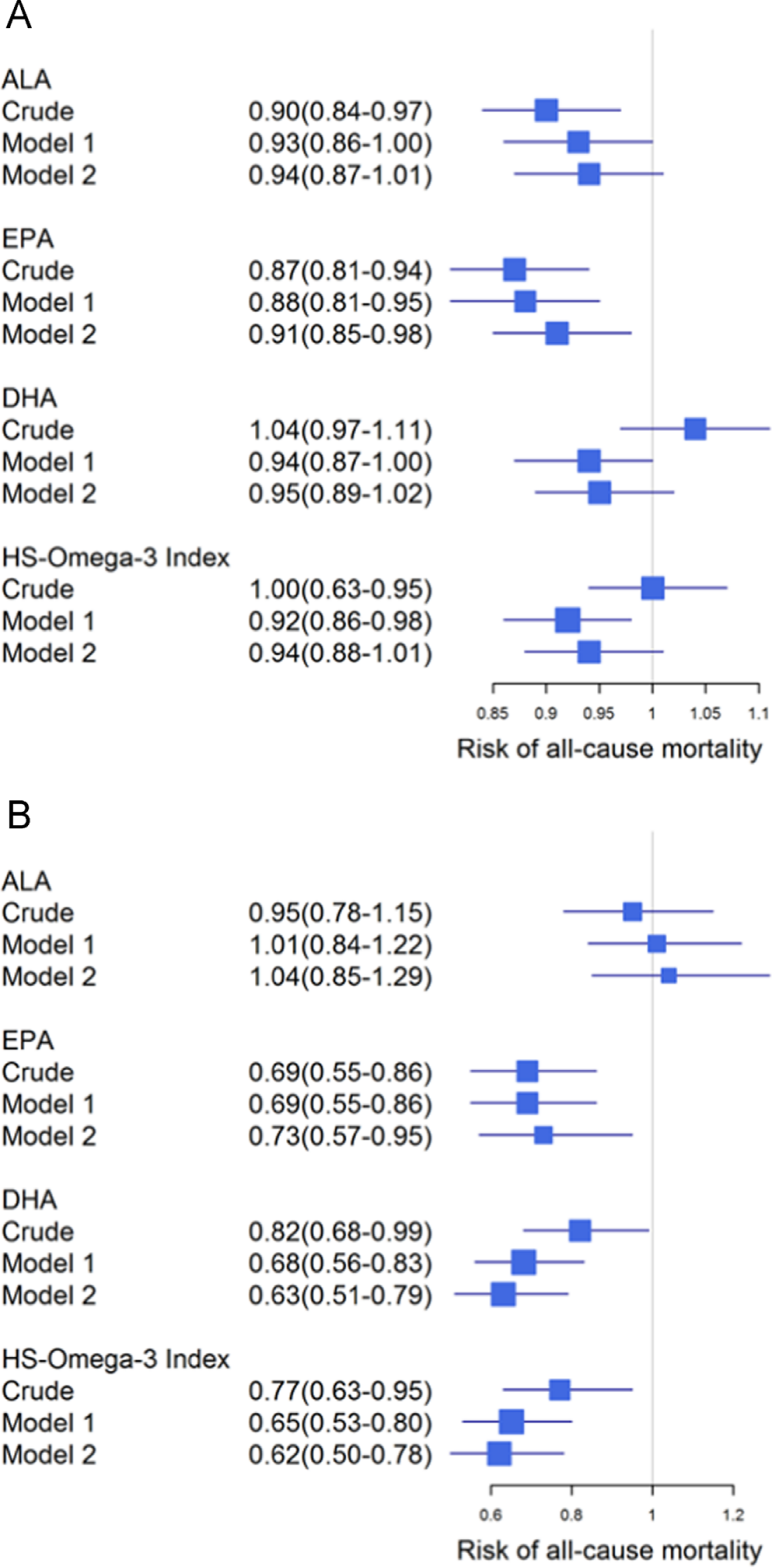
Association of omega-3 fatty acids with all-cause mortality stratified by CAD status. Hazard ratios and 95% CI were calculated for ALA, EPA, DHA and the HS-Omega-3 Index using Cox regression in patients with (A) and without (B) CAD at baseline. Model 1: adjusted for age and gender; model 2: additionally adjusted for BMI, LDL-C, HDL-C, logTG, hypertension, diabetes, smoking, alcohol intake, physical exercise and lipid lowering therapy. ALA: α-linolenic acid; EPA: eicosapentaenoic acid; DHA: docosahexaenoic acid; TG: triglycerides.

**Fig. 7:**
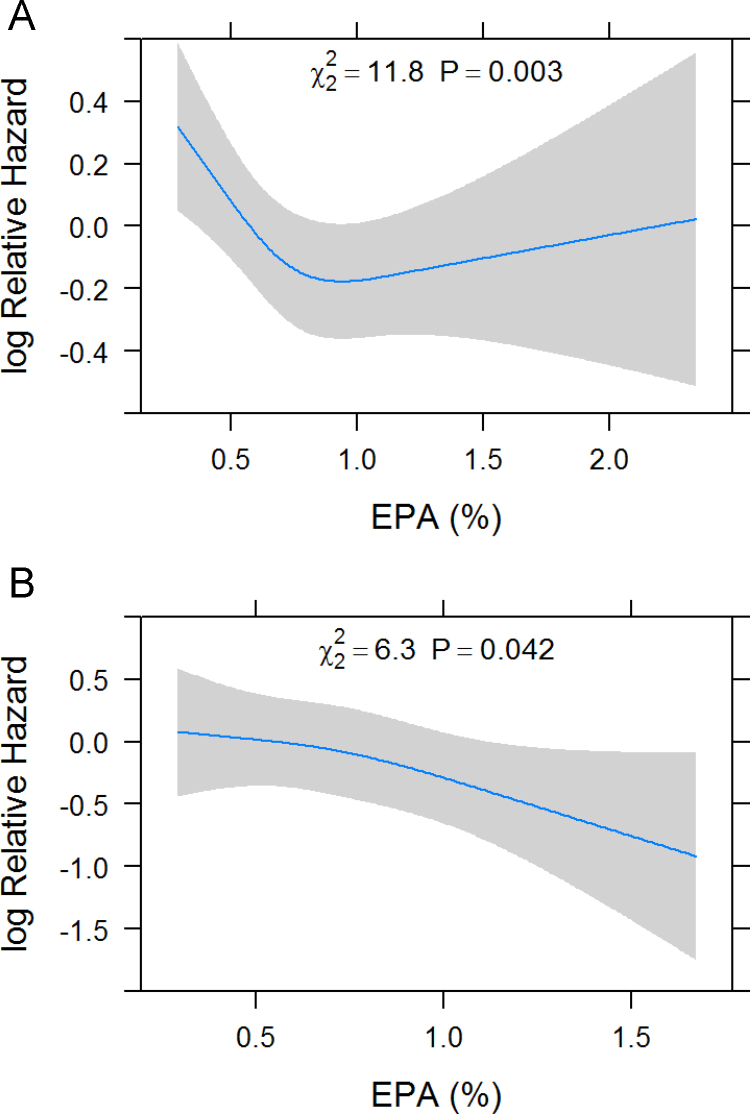
Hazard ratio plots showing the relationship between EPA proportion and all-cause mortality. Results are shown for men (A) and women (B). In a Cox regression model including age, sex, BMI, LDL-C, HDL-C, TG, smoking, alcohol intake, diabetes mellitus, hypertension, physical exercise, lipid lowering therapy and EPA, the EPA proportion was modeled as restricted cubic spline with three knots and plotted against the log hazard. EPA: eicosapentaenoic acid.

The data presented in this paper includes Figures and Tables that show the results of gender and subgroup stratified distribution of omega-3 fatty acids proportions in the LURIC study as well as multivariate adjusted analyses of their association with all-cause and cause-specific mortality that extend the results reported in [1] (Fig. 1, Fig. 2, Fig. 3, Fig. 4, Fig. 5, Fig. 6, Fig. 7 and Fig. 1, Fig. 2, Table 3, Table 4, Table 5, Table 6).

## 2 Experimental design, materials and methods

### 2.1 Subjects

The LURIC study consists of 3316 Caucasians with an indication for coronary angiography that were between 1997 and 2000 at the Ludwigshafen Heart Center in South-West Germany. A detailed description of the study can be found in Winkelmann et al. [3]. The study was approved by the ׳Landesärztekammer׳ Ethics Committee of the Rheinland-Pfalz state in Germany. Informed written consent was obtained from all participants.

### 2.2 Laboratory procedures

The fatty acid composition of erythrocyte membranes was analyzed using the HS-Omega-3 Index® methodology as described in [1], [2]. Results are given as a percentage of total identified fatty acids after response factor correction.

### 2.3 Definition of clinical variables and endpoints

The definition of clinical endpoints is detailed in [1], [3]. The 2012 CKD-EPI eGFRcreat-cys equation was used to estimate the glomerular filtration rate [4]. Information on vital status of study participants was requested from local registries. Cardiovascular mortality included sudden cardiac death (*n*=254, 7.8%), fatal myocardial infarction (*n*=104, 3.2%), death due to congestive heart failure (*n*=148, 4.5%), death after intervention to treat CAD (*n*=26, 0.8%), fatal stroke (*n*=60, 1.8%), and other causes of death due to CAD (*n*=19, 0.6%).

### 2.4 Statistical analyses

We present the mean and the standard deviation of continuous data when normally distributed and the median and 25th and 75th percentile for variables with a skewed distribution. Categorical data are presented as percentages. ANOVA was used to compare continuous variables between groups (variables with a skewed distribution were log-transformed before entering analysis) and the chi-square test was used for categorical variables. The association of omega-3 fatty acid levels in tertiles or as *Z*-transformed values with mortality was assessed by Cox proportional hazard regression with the same adjustments as in [1]. Examination of scaled Schoenfeld residuals provided no evidence for a violation of the proportional hazard assumption. All tests were two-sided and a *p* value <0.05 was considered statistically significant. SPSS v22.0 (IBM, Ehningen, Germany) and R v3.2.3 (http://www.r-project.org) were used for all analyses. The R-package ׳rms׳ was used for the generation of hazard ratio plots.

## Grant support

LURIC was supported by the 7th Framework Program RiskyCAD (Grant agreement number 305739) of the European Union and by the INTERREG IV Oberrhein Program (Grant agreement number A28) (Project A28, Genetic mechanisms of cardiovascular diseases). The work of W.M., S.L. and M.E.K. is supported as part of the Competence Cluster of Nutrition and Cardiovascular Health (nutriCARD) which is funded by the German Federal Ministry of Education and Research (Grant agreement number 01EA1411A). The funding sources had no involvement in study design, in the collection, analysis and interpretation of data, in the writing of the report and in the decision to submit the article for publication

## Figures and Tables

**Table 1:** Study characteristics stratified by gender (mean±SD or median and 25th–75th percentile).

	**Male**	**Female**	*p*^a^
	*N*=2270	*N*=989	
Age (years)	61.8 (64.7)	64.7 (10.2)	<0.001
BMI (kg/m^2^)	27.6 (3.78)	27.3 (4.66)	0.103
LDL-C (mmol/L)	2.95 (0.84)	3.16 (0.98)	<0.001
HDL-C (mmol/L)	0.97 (0.26)	1.08 (0.31)	<0.001
TG (mmol/L)	1.67 (1.24–2.28)	1.63 (1.20–2.26)	0.016
Systolic blood pressure (mmHg)	141 (23.2)	141 (24.6)	0.870
Diastolic blood pressure (mmHg)	81.7 (11.5)	79.5 (11.4)	<0.001
Fasting glucose (mmol/L)	5.70 (5.23–6.59)	5.64 (5.14–6.56)	0.004
hsCRP (mg/L)	3.28 (1.24–8.63)	3.58 (1.45–8.63)	0.107
NT-proBNP (ng/ml)	286 (98.0–858)	313 (132–878)	0.002
RBC ALA (%)	0.118 (0.046)	0.123 (0.045)	0.001
RBC EPA (%)	0.77 (0.32)	0.75 (0.28)	0.053
RBC DPA (%)	2.47 (0.36)	2.36 (0.336)	<0.001
RBC DHA (%)	5.05 (1.12)	5.12 (1.02)	0.102
RBC HS-Omega-3 Index (%)	5.82 (1.33)	5.86 (1.19)	0.365
CAD (%)	83.6	64.7	<0.001
Diabetes mellitus (%)	40.1	39.0	0.586
Hypertension (%)	71.4	76.2	0.005
Arrhythmia (%)	15.4	13.1	0.930
Smoking (active/ex/never) (%)	26.2/50.7/23.0	17.2/19.0/63.8	<0.001
Lipid lowering therapy (%)	50.6	43.9	<0.001

a *t*-test for continuous variables (non-normally distributed variables were log transformed before entering analysis), *χ*^2^ test for categorical variables.

**Table 2:** Study characteristics according to tertiles of α-linoleic acid (mean±SD or median and 25th–75th percentile).

	**ALA (% of total fatty acids)**			
	≤0.10	0.11–0.13	≥0.14	*p*^a^
	*N*=1384	*N*=952	*N*=923	
Age (years)	63.5±10.6	62.8±10.8	61.5±10.4	<0.001
Sex (% male)	72.7	69.3	65.4	0.001
BMI (kg/m^2^)	27.6±3.99	27.4±4.07	27.5±4.18	0.455
LDL-C (mg/dl)	114±32.1	117±35.1	120±36.4	<0.001
HDL-C (mg/dl)	37.4±10.1	39.3±11.2	40.5±11.4	<0.001
TG (mg/dl)	136 (103–185)	144 (107–197)	166 (120–236)	<0.001
LDL radius (nm)	8.28±0.20	8.28±0.23	8.28±0.24	0.815
Systolic blood pressure (mmHg)	141±23.8	141±23.4	141±23.6	0.967
Diastolic blood pressure (mmHg)	80.1±11.6	81.2±11.6	82.1±11.1	<0.001
Fasting glucose (mg/dl)	102 (92.7–118)	103 (94.0–118)	103 (95.0–119)	0.104
hsCRP (mg/L)	4.28 (1.50–10.1)	3.28 (1.33–8.35)	2.60 (1.12–6.18)	<0.001
NT-proBNP (ng/ml)	367 (139–1029)	278 (100–848)	214 (86.0–604)	<0.001
CAD (%)	82.5	75.4	73.5	<0.001
Diabetes (%)	40.9	39.9	37.9	0.357
Hypertension (%)	73.3	72.2	72.9	0.821
Arrhythmia (%)	15.0	16.0	12.9	0.147
Smoking (active/ex/never) (%)	22.9/44.6/32.5	23.0/36.9/40.1	24.8/40.1/35.1	0.001
Lipid lowering therapy (%)	53.8	47.4	42.0	<0.001

a ANOVA for continuous variables (non-normally distributed variables were log transformed before entering analysis), *χ*^2^ test for categorical variables.

**Table 3:** Study characteristics according to tertiles of eicosapentaenoic acid (mean±SD or median and 25th–75th percentile).

	**EPA (% of total fatty acids)**			
	≤0.60	0.61–0.62	≥0.83	*p*^a^
	*n*=1096	*n*=1115	*n*=1048	
Age (years)	63.0±10.9	62.3±11.0	62.8±9.9	0.261
Sex (% male)	68.8	69.6	70.6	0.658
BMI (kg/m^2^)	27.4±4.0	27.5±4.2	27.5±4.0	0.830
LDL-C (mg/dl)	112.1±32.4	116.8±35.1	120.8±34.8	<0.001
HDL-C (mg/dl)	37.0±10.1	38.4±10.2	41.1±11.8	<0.001
TG (mg/dl)	153(113–210)	150(112–206)	136(102–187)	<0.001
LDL radius (nm)	8.27±0.21	8.28±0.23	8.30±0.22	0.015
Systolic blood pressure (mmHg)	141±23.6	140±23.9	142±23.4	0.295
Diastolic blood pressure (mmHg)	80.7±11.5	80.7±11.6	81.7±11.3	0.076
Fasting glucose (mg/dl)	102(93.7–120)	102(93.6–119)	102(93.6–116)	0.279
hsCRP (mg/L)	3.74(1.38–9.30)	3.35(1.35–8.56)	3.02(1.18–7.99)	0.005
NT-proBNP (ng/ml)	352(136–1118)	274(97.0–773)	248(98.0–756)	<0.001
CAD (%)	78.7	78.5	76.3	0.341
Diabetes (%)	43.2	39.1	36.8	0.009
Hypertension (%)	73.9	72.4	72.3	0.642
Arrhythmia (%)	15.5	15	13.6	0.446
Smoking (active/ex/never) (%)	24.4/41.8/33.9	24.2/38.7/37.0	21.8/42.7/35.5	0.210
Lipid lowering therapy (%)	49.5	49.7	46.4	0.224

a ANOVA for continuous variables (non-normally distributed variables were log transformed before entering analysis), *χ*^2^ test for categorical variables.

**Table 4:** Study characteristics according to tertiles of docosahexaenoic acid (mean±SD or median and 25th–75th percentile).

	**DHA(% of total fatty acids)**			
	≤4.53	4.54–5.47	≥5.48	*p*^a^
	*n*=1090	*n*=1084	*n*=1085	
Age (years)	60.1±11.2	62.6±10.5	65.3±9.46	<0.001
Sex (% male)	72.4	69.3	67.3	0.033
BMI (kg/m^2^)	27.2±4.1	27.7±4.0	27.4±4.1	0.017
LDL-C (mg/dl)	117.4±33.2	117.2±34.6	114.8±35.0	0.145
HDL-C (mg/dl)	38.9±10.8	38.4±10.4	39.1±11.4	0.318
TG (mg/dl)	156(112–216)	149(113–203)	135(102–187)	<0.001
LDL radius (nm)	8.27±0.21	8.28±0.23	8.29±0.22	0.114
Systolic blood pressure (mmHg)	140±23.6	141±23.8	142±23.4	0.237
Diastolic blood pressure (mmHg)	81.3±11.7	81.0±11.5	80.7±11.2	0.391
Fasting glucose (mg/dl)	101(93.2–115)	104(94.4–120.9)	102(93.4–119)	0.122
hsCRP (mg/L)	3.32(1.24–7.91)	3.57(1.38–9.24)	3.34(1.23–8.54)	0.107
NT-proBNP (ng/ml)	276(95.8–757)	292(105–934)	311(116–921)	0.013
CAD (%)	75.2	78.5	79.9	0.026
Diabetes (%)	35.2	42.3	41.8	0.001
Hypertension (%)	70.9	74.3	73.5	0.187
Arrhythmia (%)	13.5	13.8	16.9	0.051
Smoking (active/ex/never) (%)	33.0/36.2/30.7	23.5/42.4/34.0	13.8/44.5/41.7	<0.001
Lipid lowering therapy (%)	47.0	48.5	50.2	0.315

a ANOVA for continuous variables (non-normally distributed variables were log transformed before entering analysis), *χ*^2^ test for categorical variables.

**Table 5:** Comparison of Cox regression analyses modeling EPA proportion as linear term or as restricted cubic spline adjusted for risk factors (model 2).

	**loglik**	**Chisq**	**Df**	***p***
*Combined*				
Spline model	−7151.0			
Linear model	−7153.5	4.9778	1	0.02567
				
*Men*				
Spline model	−5072.7			
Linear model	−5076.3	7.1767	1	0.007386
				
*Women*				
Spline model	−1519.7			
Linear model	−1519.9	0.3817	1	0.5367

**Table 6:** Association between omega-3 fatty acids and cause-specific death.

		**Death due to heart failure**		**Sudden cardiac death**	
		HR (95% CI)	*p*	HR (95% CI)	*p*
***a-Linolenic acid***					
Model 1	1st (≤0.10%)	1^reference^		1^reference^	
	2nd (0.11–0.13%)	0.75 (0.50–1.12)	0.154	0.77 (0.57–1.04)	0.088
	3rd (≥0.14%)	0.96 (0.65–1.42)	0.838	0.96 (0.71–1.29)	0.761
*p*_Trend_			0.346		0.222
Model 2	1st (≤0.10%)	1^reference^		1^reference^	
	2nd (0.11–0.13%)	0.79 (0.53–1.19)	0.258	0.80 (0.59–1.09)	0.159
	3rd (≥0.14%)	1.05 (0.69–1.58)	0.831	1.05 (0.77–1.44)	0.764
*p*_Trend_			0.422		0.252
***Eicosapentaenoic acid***					
Model 1	1st (≤0.60%)	1^reference^		1^reference^	
	2nd (0.61–0.82%)	0.71 (0.49–1.04)	0.077	0.93 (0.69–1.24)	0.600
	3rd (≥0.83%)	0.62 (0.42–0.93)	0.020	0.71 (0.52–0.98)	0.034
*p*_Trend_			0.044		0.094
Model 2	1st (≤0.60%)	1^reference^		1^reference^	
	2nd (0.61–0.82%)	0.77 (0.52–1.13)	0.184	0.98 (0.73–1.31)	0.886
	3rd (≥0.83%)	0.69 (0.46–1.04)	0.076	0.75 (0.54–1.04)	0.081
*p*_Trend_			0.167		0.165
***Docosahexaenoic acid***					
Model 1	1st (≤4.53%)	1^reference^		1^reference^	
	2nd (4.54–5.47%)	0.80 (0.53–1.20)	0.273	1.03 (0.76–1.41)	0.833
	3rd (≥5.48%)	0.85 (0.57–1.26)	0.411	0.94 (0.69–1.28)	0.700
*p*_Trend_			0.525		0.815
Model 2	1st (≤4.53%)	1^reference^		1^reference^	
	2nd (4.54–5.47%)	0.76 (0.50–1.15)	0.197	0.95 (0.69–1.30)	0.741
	3rd (≥5.48%)	0.84 (0.56–1.26)	0.388	0.91 (0.66–1.26)	0.574
*p*_Trend_			0.425		0.853
***HS-Omega-3 Index***					
Model 1	1st (≤5.19%)	1^reference^		1^reference^	
	2nd (5.20–6.24%)	0.74 (0.49–1.10)	0.137	1.11 (0.82–1.50)	0.517
	3rd (≥6.25%)	0.77 (0.52–1.13)	0.182	0.85 (0.62–1.16)	0.305
*p*_Trend_			0.260		0.212
Model 2	1st (≤5.19%)	1^reference^		1^reference^	
	2nd (5.20–6.24%)	0.73 (0.49–1.10)	0.133	1.04 (0.76–1.41)	0.822
	3rd (≥6.25%)	0.78 (0.52–1.16)	0.221	0.82 (0.60–1.14)	0.242
*p*_Trend_			0.278		0.303

Model 1: adjusted for age and gender.

Model 2: additionally adjusted for LDL-C, HDL-C, logTG, BMI, hypertension, diabetes mellitus, smoking, alcohol intake, physical exercise and lipid lowering therapy.
