# Supplementary material for: Data on gender and subgroup specific analyses of omega-3 fatty acids in the Ludwigshafen Risk and Cardiovascular Health Study
Source: Data Brief. 2016 Aug 3;8:1311–21. doi: 10.1016/j.dib.2016.07.051 (PMC4990639; doi:10.1016/j.dib.2016.07.051)
Supplement: Supplementary file 1 — Supplementary material [file mmc1.doc]

Conflict of Interest

C.v.S. has founded Omegametrix GmbH, a laboratory performing fatty acid analysis, which analyzed LURIC samples free of charge. W.M. is employed with synlab Holding Deutschland GmbH, a medical diagnostics service provider. G.D., S.L. and M.E.K. declare no conflict of interest.
